
JOURNAL INFORMATION
==============================
NLM Title Abbreviation: Gastroenterol Rep (Oxf)
Journal ID: Gastroenterol Rep (Oxf)
NLM Journal ID: 101620508
Journal ID: gastro

Gastroenterology Report

EISSN: 2052-0034
Publisher: Oxford University Press

ARTICLE INFORMATION
==============================
PMCID: PMC6225816
DOI: 10.1093/gastro/goy043
Article ID: goy043
Article version: 1
Subjects: Chinese Abstracts

Chinese Abstracts – Volume 6 Issue 4*

This article abstract was originally published in English. This translation has not been verified and should not be relied upon—it is provided for reference purposes only. The Publishers have not checked this translation and accept no liability for completeness or accuracy of this translation or the use of this translation for whatever purpose. This translation may be incomplete and inaccurate in whole or in part. If you need to rely upon a translation of this abstract, a professional human translator should be engaged to supply an accurate translation of the original English. When referencing articles from this journal, please always refer to the original English version, rather than a translated equivalent.

Print publication date: 2018 Nov
Electronic publication date: 2018 Nov 9
Volume: 6
Issue: 4
First page: C1
Last page: C3
Copyright: © The Author(s) 2018. Published by Oxford University Press and Sixth Affiliated Hospital of Sun Yat-Sen University
Copyright year: 2018
License: This is an Open Access article distributed under the terms of the Creative Commons Attribution License (http://creativecommons.org/licenses/by/4.0/), which permits unrestricted reuse, distribution, and reproduction in any medium, provided the original work is properly cited.
License URL: https://creativecommons.org/licenses/by/4.0/

Page count: 3

==============================
Gastroenterology Report, 2018, 1–3
doi: 10.1093/gastro/goy043

ABSTRACTS

### Chinese Abstracts – Volume 6 Issue 4*

### Editorial（述评）

### Patient-derived organoids: a promising model for personalized cancer treatment
### 患者来源的类器官：一种有前景的个体化癌症治疗模式

### Review （综述）

### Diagnosis and management of floppy pouch complex
### 软袋疾病群的诊断和治疗

[摘要] 全结直肠切除、回肠储袋肛管吻合术已经成为顽固性溃疡性结肠炎、肠炎相关不典型增生或家庭性腺瘤性息肉病的外科治疗术式。但术后各种储袋疾病及相关并发症也时有发生。软袋疾病群（Floppy pouch complex）定义为经储袋镜、泛影葡胺储袋造影或排粪造影发现的储袋脱垂、输入襻综合征、肠膨出或储袋冗长折叠， 其共同的临床表现包括排便困难、腹胀、腹痛、排便费力或排便不尽感。每种软袋疾病都有其独有的内镜、影像学及动力学表现；可根据不同的病因选择不同的治疗方式。

### Management of chronic hepatitis B during pregnancy
### 孕期慢性乙型肝炎的治疗

[摘要] 慢性乙型肝炎是一种全球性疾病，也是肝硬化和肝癌的主要发生原因。尽管对于乙肝孕妇进行免疫预防， 但仍有至少10%的胎儿会通过母婴传播出现病毒血症。对于乙肝孕妇的治疗决策必须要兼顾治疗获益和母婴的安全性。我们总结了当前慢性乙型肝炎的治疗方法， 并着重探讨了孕期乙肝的治疗， 并基于循证证据提出了对乙肝病毒母婴垂直传播的预防策略。

### Original article （论著）

### Colorectal cancer resection rates in patients with inflammatory bowel disease: a population-based study
### 炎症性肠病患者中结直肠癌患者的切除率：基于人群的研究

[摘要] 背景与目的：炎症性肠病（IBD）患者结直肠癌的发生风险升高。本研究旨在分析美国IBD相关结直肠癌手术切除率的变化趋势。

方法：我们收集了美国住院样本数据库1995－2012年间的相关病例资料。采用多因素Joinpoint回归模型分析年龄标化的IBD相关结直肠癌的手术切除率的时间变化趋势。主要结局指标是IBD相关结直肠癌的手术切除率。

结果：共纳入1995－2012年间3, 597, 168例IBD出院病例, 其中275, 479例接受了结直肠癌切除手术。IBD人群中的结直肠癌切除率从1995年至2012年显著下降, 各个年龄组的年切除例数均显著下降, 18-39岁组、40-49岁组、50-74岁组和75岁以上组每100， 000例IBD患者中结直肠癌年切除例数从1995年至2012年分别减少393、359、293和159例（均$P < 0.001$）；其中， 近端结直肠癌年切除例数分别减少149、130、95和50例（均$P < 0.001$）, 远端结直肠癌年切除例数分别减少104、123、123和82例（均$P < 0.001$）。多因素Poisson回归分析显示, 校正年龄和性别因素后, 结直肠癌切除率从1995－2012年间年均下降了3.9%。

结论：从1995－2012年间， IBD患者中的结直肠癌切除率逐年下降, 且近端和远端结直肠癌切除率均呈现下降趋势。这一人群水平的下降趋势反应了美国IBD相关结直肠癌发病率的下降。

### Prognostic and predictive value of interstitial cells of Cajal populations following stapled transanal rectal resection (STARR) in patients with obstructed defecation syndrome
### 肠道Cajal细胞数量对行吻合器经肛直肠切除术（STARR）的梗阻性排便综合征患者的预后和预测价值

[摘要] 目的：本研究旨在评估吻合器经肛直肠切除术（STARR）治疗梗阻性排便综合征（ODS）的功能学结果, 并探讨肠道Cajal细胞（ICC）数量与STARR手术疗效的关系。

方法：将50例行STARR手术的ODS患者的全层直肠标本, 采用免疫组化方法检测直肠样本中的ICC细胞数量, 并取20例来自直肠癌癌旁组织的直肠样本作为对照。收集排粪造影和直肠测压的功能学参数。

结果：无论是在黏膜下层表面（SM）、肌纤维（IM）还是环形肌与纵形肌的肌间隙（MY）中, 与对照标本相比, ODS直肠标本中的ICC细胞数量均显著减少（均$P < 0.05$）。术前克利夫兰便秘评分（CCS）为24.2 ± 4.1, 术后1、2、3、4和5年CCS评分均显著下降（均$P < 0.05$）。术后3年，58.3%（28/48）的患者疗效满意（CCA ≤ 10）。单因素分析显示， 术前需手辅助排便（$P = 0.017$）、ICC-MY细胞数量减少（$P = 0.067$）、直肠感觉阈值增加（$P = 0.073$）的ODS患者，术后功能学结果

*This article abstract was originally published in English. This translation has not been verified and should not be relied upon—it is provided for reference purposes only. The Publishers have not checked this translation and accept no liability for completeness or accuracy of this translation or the use of this translation for whatever purpose. This translation may be incomplete and inaccurate in whole or in part. If you need to rely upon a translation of this abstract, a professional human translator should be engaged to supply an accurate translation of the original English. When referencing articles from this journal, please always refer to the original English version, rather than a translated equivalent.
本文摘要最初是以英文发表。中文摘译未获认证，谨供参考。出版社没有对中文摘译进行核验，不负责译文的完整性和准确性，也不会将其用以其他目的。中文摘译可能会出现部分不完整或不准确的情况。如果你需要准确获悉摘要，最好能请一位专业译者针对英文原文进行翻译。阅读杂志文章，请参考英文原文，不推荐中文翻译。

不良。多因素分析证实，ICC-MY细胞数量减少是术后不良功能学结果的独立预测因素（OR = 0.097, 95% CI: 0.012-0.766）。

结论：STARR手术治疗ODS总体疗效满意，但随时间推移，肛门功能会有所恶化。直肠标本中ICC细胞数量减少的患者术后肛门功能不良，提示术前对直肠进行全层组织学评估或可预测STARR手术的功能学结局。

### Is adjuvant chemotherapy necessary for patients with ypT0–2N0 rectal cancer treated with neoadjuvant chemoradiotherapy and curative surgery?

### 对于新辅助放化疗加根治性手术的 ypT0－2N0 直肠癌患者，辅助化疗是必要的吗？

[摘要] 背景与目的：辅助化疗能否令新辅助放化疗加根治性手术的直肠癌患者受益仍存在争议， 尤其是对于那些新辅助放化疗反应良好者。本回顾性研究旨在明确辅助化疗能否给新辅助放化疗加根治性手术的ypT0-2N0直肠癌患者带来肿瘤学获益。

方法：回顾性收集2005－2014年间中国医学科学院肿瘤医院接受新辅助放化疗加根治性手术处理的ypT0-2N0直肠癌患者。将术后接受与不接受辅助化疗的两组患者进行预后比较。

结果：收集到110例患者的临床病理资料，其中1例患者因缺乏随访资料而删去。最终纳入研究的109例患者中， 58例（53.2%）接受了辅助化疗（化疗组），51例（46.8%）未辅助化疗（未化疗组）。中位随访时间为50个月。两组患者的5年总生存率（OS）和5年无复发生存率（RFS）的差异均无统计学意义（5年OS: 92.1% vs. 86.3%, $P = 0.375$; 5年RFS: 80.9% vs. 74.7%, $P = 0.534$）。分层分析结果显示，无论是ypT0N0还是ypT1-2N0患者，化疗组与非化疗组之间的5年OS和5年RFS的差异均无统计学意义（均$P > 0.05$）。

结论：对于接受新辅助放化疗和根治性手术治疗的ypT0－2N0直肠癌患者， 辅助化疗并不能显著改善其预后。但该结论还有待于更大样本量的前瞻性临床随机对照试验来进行验证。

### Well-controlled autoimmune hepatitis treatment withdrawal may be safely accomplished without liver-biopsy guidance

### 控制良好的自身免疫性肝炎在终止治疗前不进行肝脏活检也是同样安全的

[摘要] 背景：自身免疫性肝炎在终止治疗后可出现复发，尤其是在那些肝酶恢复正常但并未取得组织学缓解的患者。欧洲肝脏研究协会（EASL）和美国肝病研究协会（AASLD）的指南均推荐在终止治疗前要进行肝脏活检。本研究的目的是评估那些经免疫抑制治疗疾病控制良好的成人患者在终止治疗后的临床结局，无论其在终止治疗前有没有进行肝活检。

方法：本研究为回顾性观察研究， 研究对象是经组织学证实的成人自身免疫性肝炎患者，其接受了2年免疫抑制治疗，且在终止治疗前6个月内天冬氨酸转氨酶（AST）和丙氨酸转氨酶（ALT）一直维持或接近正常水平。排除青少年自身免疫性肝炎、既往曾接受过治疗或采用了激素和硫唑嘌呤之外药物治疗的患者。主要结局指标是终止治疗后1年复发率。

结果：满足纳入标准的34例连续病例纳入研究，其中24例在终止治疗前未行肝活检， 10例行肝活检。两组一般资料、免疫抑制剂使用情况、治疗前肝硬化程度和肝酶水平的差异均无统计学意义。25%的患者在终止治疗后1年内出现肝酶升高， 但未行肝活检组与行肝活检组1年复发率的差异并无统计学意义（20.8% vs. 30.0%, $P = 0.57$）。

结论：经2年免疫抑制治疗疗效甚佳的成人自身免疫性肝炎患者， 在终止治疗前无需行肝活检。

### Ileal transposition rapidly improves glucose tolerance and gradually improves insulin resistance in non-obese type 2 diabetic rats

### 空肠间置术能快速改善非肥胖2型糖尿病大鼠的糖耐量并缓慢改善胰岛素抵抗

[摘要] 背景：许多研究已证实，回肠间置术能改善2型糖尿病（T2DM），同时胰高血糖素样肽-1（GLP-1）水平上升。我们采用糖尿病大鼠模型进行研究，评估回肠间置术对糖代谢的影响及其机制。

方法：20只GK大鼠被随机分为回肠间置组和假手术组。分别于术前及术后1、4、8、16、24周检测大鼠体重、进食量、空腹血糖（FPG）、空腹胰岛素（F-ins）、口服葡萄糖耐量试验（OGTT）和GLP-1水平。计算稳态模型胰岛素抵抗指数（HOMA-IR）和OGTT曲线下面积（AUC）。术后24周处死大鼠，检测胰腺和回肠组织中GLP-1受体（GLP-1R）的表达水平。

结果：与假手术组相比， 回肠间置组大鼠术后4、8、16和24周血GLP-1水平更高（均$P < 0.05$），术后8、16和24周FPG、F-ins和HOMA-IR水平更低（均$P < 0.05$）。与术前相比，回肠间置组术后各个时间点均可观察到血GLP-1、AUC和FPG水平的下降（均$P < 0.05$）；术后8、16和24周F-ins和HOMA-IR也显著下降（均$P < 0.05$）。术后24周，无论是胰腺还是回肠组织，回肠间置组大鼠的GLP-1R表达水平均显著高于假手术组（均$P < 0.05$）。

结论：回肠间置可改善GK大鼠的糖代谢， 且不会导致体重的下降和进食量的减少，这可能与GLP-1水平升高有关。而后期胰岛素抵抗的改善和胰岛素水平的下降可能并不直接受益于GLP-1水平的升高。

### The role of visceral adipose tissue on improvement in insulin sensitivity following Roux-en-Y gastric bypass: a study in Chinese diabetic patients with mild and central obesity

### 内脏脂肪组织对于Roux-en-Y胃旁路手术后胰岛素敏感性改善的影响：一项对伴有轻度中心性肥胖的中国糖尿病患者的研究

[摘要] 背景：大多中国2型糖尿病（T2DM）患者都有一个轻度、中心性肥胖。中心性肥胖常合并胰岛素抵抗。本研究目的是评估腹部脂肪组织对合并轻度中心性肥胖的中国糖尿病患者Roux-en-Y胃旁路手术（RYGB）后胰岛素敏感性改善的影响。

方法：17例T2DM患者拟行腹腔镜RYGB手术， 平均体质指数30.3 kg/m$^2$。术前和术后3个月行高胰岛素-正葡萄糖钳夹和双能X线吸收检测。主要结局指标是胰岛素敏感性与腹部脂肪组织的关系， 后者包括术前和术后3个月检测的内脏脂肪组织（VAT）和皮下脂肪组织（SAT）。

结果：外周胰岛素敏感指标， 包括葡萄糖代谢清除率（M值）和葡萄糖输注率，均于RYGB术后显著增加，人体脂肪总量、VAT重量和SAT重量则显著下降。术前M值与VAT重量呈显著负相关（$r = -0.57$, $P = 0.02$），但与SAT重量无关。RYGB术后M值的变化值与VAT变化值（$r = -0.59$, $P = 0.01$）、VAT重量占比（$r = -0.66$, $P < 0.01$）、VAT面积（$r = -0.56$, $P = 0.02$）及VAT面积占比（$r = -0.57$, $P = 0.02$）均呈显著负相关。

结论：合并轻度中心性肥胖的中国T2DM患者RYGB手术后， 其外周胰岛素敏感性的增加与VAT的减少具有显著关联。RYGB术后， VAT和SAT均显著下降，同时胰岛素敏感明显改善。VAT重量或可作为胃旁路术的一个适应证，用于手术病例的选择。

### Serum zinc level is associated with liver dysfunction caused by white smoke inhalation

### 血锌水平与白烟吸引引起的肝损伤有关

[摘要] 背景：在作战中暴露在白烟弹下会导致器官损伤，但这种情况并不常见也鲜见报道。本研究目的是观察吸入白烟后血锌水平是否与肝功能存在相关性。

方法：15例具有白烟暴露史的患者纳入本研究，分析临床表现、血锌水平与及肝功能之间的关系。

结果：相对11例轻度患者，4例中重度患者血锌水平显著增高。4例中重度患者均出现延迟肝功能损害，丙氨酸转氨酶增高。此外，3例中重度患者血浆透明质酸浓度增高。但没有检测到六氯乙烷和三氯丙烷。

结论：吸入白烟的患者，其血锌水平与肝损伤严重程度具有相关性。

### Case report （病例报告）

### Budd-Chiari syndrome associated with coeliac disease: case report and literature review

### 乳糜泻相关的Budd-Chiari综合征：病例报告及文献复习

**A rare case of plexiform angiomyxoid myofibroblastic tumor in the stomach which was diagnosed at the earliest stage in the literature**
文献中最早获得诊断的胃丛状血管黏液样肌纤维母细胞瘤

### Study protocol （研究方案）

**Morbidity and mortality of elderly patients with advanced gastric cancer after laparoscopy-assisted or open distal gastrectomy: a randomized–controlled trial**
腹腔镜辅助对比开腹远端胃切除术治疗老年进展期胃癌的并发症发生率和病死率：随机对照试验

### Corrigendum（更正启示）

goy035
goy037
